# Preventing infection after synthetic expander implantation in patients undergoing breast reconstruction

**DOI:** 10.20407/fmj.2020-029

**Published:** 2021-08-20

**Authors:** Satoko Onishi, Yoshikazu Inoue, Maki Inukai, Takayuki Okumoto

**Affiliations:** Department of Plastic and Reconstructive Surgery, School of Medicine, Fujita Health University, Toyoake, Aichi, Japan

**Keywords:** Breast reconstruction, Tissue expanders, Infection

## Abstract

**Objectives:**

Breast reconstruction using synthetic materials has increased rapidly in Japan since July 2013, when national health insurance began covering the procedure. Although synthetic material-based reconstruction of other body parts has not resulted in wounds with complications, this significant advantage is overshadowed by a risk of complications, including infection, following breast reconstruction. We therefore reviewed breast-reconstruction patients who experienced infection after implantation of synthetic materials and the countermeasures we used to address the problem.

**Methods:**

From July 2013 through December 2019, our department performed primary breast reconstructions using tissue expanders (TEs) in 106 patients and secondary breast reconstructions in 39 patients. We retrospectively reviewed these 145 patients in terms of their age, body mass index, timing of the reconstruction, presence/absence of both chemotherapy and radiation therapy before and after surgery, presence/absence of postoperative wound complications, and presence/absence of atopic dermatitis. We then evaluated whether these factors put patients at risk for postoperative TE infection.

**Results:**

Among the 145 patients who underwent reconstruction with TE, 3 (2.0%) were diagnosed with a postoperative TE infection. Our review revealed that necrosis of the skin around the surgical wound (P=0.004) and atopic dermatitis (P=0.041) were risk factors for TE infection.

**Conclusions:**

Infection following breast reconstruction with synthetic materials is a serious complication. Thus, patients requiring this surgery deserve optimal perioperative management. For those with known risk factors, a more appropriate surgical approach—e.g., using autologous tissue instead of a synthetic material—could be considered.

## Introduction

Tissue expanders (TEs) (Natrelle^®^ 133 Tissue Expander, Allergan, Dublin, Ireland) and artificial mammary glands (Natrelle^®^ 410 Breast Implant, Allergan) have been covered by national health insurance in Japan for breast reconstruction following breast cancer surgery since July 2013. The patients requesting breast reconstruction has increased since then. When mastectomy is performed, it is common practice to resect skin along with the areola and nipple, causing insufficient skin available to cover the silicone implant that will replace the mammary gland. Thus, in patients in whom the skin defect is large, a TE is implanted under the greater pectoral muscle, and physiological saline is gradually injected over several months to expand the TE and overlying tissues. Once the skin is considered sufficiently extended, the TE is replaced with a silicone implant or autologous tissue.

Not all patients have a TE implanted at the same time as their mastectomy. Depending on the patient’s wishes along with the scheduled cancer treatment, a TE may be implanted at the time of the mastectomy (primary reconstruction) or several months or years later (secondary reconstruction). To achieve favorable breast reconstruction results, skin extension using a TE lays the groundwork for reconstruction with an implant or autologous tissue, thereby playing an important role. Because TEs are synthetic, however, infection can be a significant complication. Infection of an artificial material is often difficult to eradicate, significantly reducing the patient’s satisfaction. It may also cause extended hospitalization and increase the patient’s medical costs. Accordingly, it is important to prevent infections.^1,2^ We therefore retrospectively reviewed surgical records of patients who experienced infection following TE implantation. We discuss our findings and the actions that may prevent such infections.

## Methods

This retrospective study, covering July 2013 through December 2019, included 106 patients who underwent primary breast reconstruction using TE and 39 who underwent secondary breast reconstruction during that period. Primary reconstruction is indicated for patients who want to undergo reconstruction at the same time as breast resection to avoid sensing the loss of a breast. In contrast, secondary reconstruction postpones the surgery until after completion of all cancer treatment. This choice depends on the patient’s desire.

The surgical method for primary reconstruction in this study was as follows: After mastectomy, the wound was washed with 2000 mL of physiological saline by a breast surgeon. Before transferring a patient to the Plastic Surgery Department, the surgical-site drape and equipment were replaced, and disinfection was repeated. Next, in all patients—regardless of whether they underwent primary or secondary reconstruction—a TE was implanted below the greater pectoral muscle and covered with muscle or fascia. During primary reconstruction, drains were placed both below the greater pectoral muscle and subcutaneously. When axillary dissection had been performed, another drain was placed at the axilla. For patients with secondary reconstruction, a drain was placed only below the greater pectoral muscle.

TEs range in size from 200 to 950 mL A TE was selected based on the width, height, and projection of the breast. Physiological saline was injected into the TE about a week after surgery, depending on the condition of the wound. Subsequently, 40–80 mL was injected every 1–2 weeks. Cefazolin 1 g/dose was used as prophylaxis for both primary and secondary reconstructions. It was administered intravenously immediately preoperatively and every 3 h intraoperatively. Postoperatively, it was infused twice daily for 2 days, followed by oral administration of cefaclor 750 mg/day for 5 days. Drains were removed once the amount of drainage fluid was <30 mL for two consecutive days. Infection was diagnosed if the following symptoms were observed: local inflammation including erythema and/or swelling at the wound site, fever, turbidity of drainage fluid, or increased C-reactive protein (CRP) and white blood cell (WBC) count.

Retrospective review of the following variables was conducted for all patients: age, body mass index (BMI), timing of reconstruction, presence/absence of both chemotherapy and radiation therapy before and after surgery, presence/absence of postoperative wound complications, and presence/absence of atopic dermatitis. We evaluated risk factors for postoperative TE infection. For the statistical analysis, we evaluated the patient’s age, BMI, timing of reconstruction, necrosis of the skin at the wound, and the presence/absence of atopic dermatitis using Fisher’s exact test. The presence/absence of irradiation and chemotherapy was evaluated using the χ^2^ test. A value of P<0.05 was considered to indicate statistical significance.

## Results

The patients’ mean age was 51 years (range 29–72 years) and their mean BMI was 22.7 kg/m^2^ (range 16.1–37.2 kg/m^2^). In all, 13 patients underwent chemotherapy (1 patient preoperatively, 12 patients postoperatively), and 8 underwent radiation therapy (6 preoperatively and 2 postoperatively after implant reconstruction). Six patients had a skin ulcer at the wound (all after primary reconstruction). All six ulcers were due to impaired blood flow at the wound site. Two of these six patients underwent debridement and resuturing, and four were managed conservatively. Of the two patients with atopic dermatitis, only one case was poorly controlled.

Among the 106 patients who underwent primary reconstruction with TE, 3 (2.5%) were diagnosed as having an infection. None of the 39 patients with secondary reconstruction experienced an infection.

The first patient with an infection had poorly controlled atopic dermatitis. The patient exhibited inflammatory symptoms, including erythema and swelling at the wound site, fever, WBC 7200/μL, and CRP 16.5 mg/dL on postoperative day 8. Methicillin-resistant *Staphylococcus aureus* (MRSA) was isolated from the drainage fluid. Treatment with intravenous vancomycin was unsuccessful, and the TE was subsequently removed.

In the second patient with an infection, skin around the postoperative wound had become necrotic, resulting in an ulcer. Conservative treatment with ointment was provided. Around 1 month postoperatively, the patient showed inflammatory signs due to chest erythema as well as fever, WBC count 8600/μL, and CRP 4.46 mg/dL. Under ultrasound guidance, the liquid surrounding the TE was punctured and drained, with methicillin-sensitive *Staphylococcus aureus* (MSSA) isolated. The patient’s condition improved with intravenous antibiotics, although the infection relapsed. Consequently, the TE was removed.

The third patient with an infection had a skin ulcer at the postoperative wound site. It was treated conservatively with ointment, and the wound stabilized. Chemotherapy was started 1 month postoperatively, after which inflammatory findings, including chest erythema, fever, WBC 18,400/μL, and CRP 15.71 mg/dL, were observed. Treatment with intravenous antibiotics was unsuccessful, and the implant was removed. MSSA was isolated from cultures of the wound site specimen that had been collected intraoperatively.

We then investigated the correlation of the postoperative infections in the three patients with the following variables: age, BMI, timing of reconstruction, presence/absence of both chemotherapy and radiation therapy before and after surgery, presence/absence of a postoperative wound complication, and the presence/absence of atopic dermatitis. We found that skin necrosis at the wound (P=0.004) and atopic dermatitis (P=0.041) were risk factors for postoperative TE infection (Table 1).

## Discussion

Infection is the most important complication of breast reconstruction using synthetic materials. The infection rate after TE implantation varies depending on the surgical procedure and the indication for the TE. One previous study reported an infection rate of 6.1%,^1^ whereas the Japan Oncoplastic Breast Surgery Society reported an infection rate of 2.4%.^3^ At our facility, the infection rate is 2.0%.

Several reports have addressed risk factors for these infections.^1,2,4–6^ The present study indicated that skin necrosis at the surgical wound site and atopic dermatitis, a skin barrier function abnormality, were risk factors for TE infection.

For primary reconstruction following mastectomy, necrosis of breast skin has been reported as increasing the probability of postoperative infection of synthetic materials.^4,6^ At our hospital, only six patients who underwent primary reconstruction developed skin necrosis. Among them, two patients underwent debridement and re-suturing, and two patients were successfully treated with short-term topical treatment and could complete implant reconstruction. The remaining two patients required more than a month for the wound to stabilize. During this time period, infection occurred.

Skin necrosis at the surgical wound site was observed in six patients with primary reconstruction, suggesting that procedures to protect the skin around the wound during mastectomy are essential. Additionally, if the skin necrosis advances, exposing the artificial materials, infection is almost certain. Accordingly, it is crucial to cover artificial materials with healthy muscles and muscular fascia with good blood circulation, which are resistant to infection. Moreover, if an excessive amount of physiological saline is injected into the TE intraoperatively, high tension is created at the wound area when closing the surgical site, exacerbating blood flow disturbance and increasing the risk of skin infection around the wound. Therefore, to avoid excessive tension when closing the wound, it is advisable to inject an appropriate amount of saline. In addition, if a skin complication occurs around the wound, it may be difficult to cure. Hence, an early decision to debride and re-suture is advisable. Our experience has taught us that replacing the TE as a salvage procedure and/or continuously washing the site are options. As the risk of relapse is high, however, with the consent of the patient we remove the TE and perform the reconstruction once the infection is controlled.

Although not a finding in this study, others have reported that radiation therapy and chemotherapy are risk factors.^7–11^ For patients with a history of radiation therapy, however, we consider reconstruction with autologous tissues if the irradiation occurred recently. For patients whose radiation therapy was more than a year prior, however, we assess the patient’s skin elasticity and then explain to the patient the complications associated with the use of synthetic materials. We then perform the reconstruction with an implant only when a patient still requests reconstruction with synthetic material.

For patients who require irradiation after mastectomy, it is not performed around the time of TE implantation. Instead, the patient undergoes irradiation at least 1 month after installation of the permanent implant. Rigorous skin care is provided after such irradiation. Moreover, for patients who have undergone chemotherapy before the surgery, the date of surgery is taken into consideration regarding recovery of immune function and when the patient’s systemic condition has stabilized. Among our patients who had been given adjuvant chemotherapy, we experienced one case of postoperative infection. When a skin ulcer at the margin of the surgical wound has not healed quickly, debridement and re-suturing to accelerate wound healing may be considered.

Concerning the selection of antibiotics, the most common causative bacterium with artificial materials is *Staphylococcus aureus*,^1,12^ for which we administered cefazolin. The appropriate timing of the antibiotics was also considered and whether it would be effective for MSSA infections. Both of our patients experienced skin necrosis for more than a month, and antibiotics were not used during that time. At the initial stage of the infection, mild redness and fever had been overlooked, and antibiotics were not given in a timely manner. With progression of the infection, however, and with further symptoms (e.g., fever), antibiotics were again administered. The resumption of antibiotics, however, appeared to be too late.

Another patient who experienced MRSA infection had uncontrolled atopic dermatitis. MRSA was detected in the drainage fluid. This patient did not improve despite intravenous infusion of vancomycin, which was considered too late. Atopic dermatitis (a skin barrier dysfunction) tends to be associated with skin MRSA.^13–15^ The reported salvage rate after MRSA infection is low.^6,12,16^ Thus, for patients with poorly controlled atopic dermatitis in whom skin MRSA is present, rigorous preoperative skin care is needed, and skin cultures must be prepared. Then, depending on the results, anti-MRSA drugs should be administered preemptively.

We offer the following precautions that, based on our experience, we believe are essential for avoiding infections.

1.  A meticulously clean surgical procedure is essential, along with use of protective procedures for skin around the wound at mastectomy and vigorous hemostasis.
2.  The TE should be covered as much as possible with muscle and fascia.
3.  Drains should be placed at appropriate locations and frequent drainage conducted.
4.  The amount of physiological saline during surgery should be adjusted to prevent wound disruption due to necrosis.
5.  In case of postoperative necrosis at the wound site, early debridement and resuturing should be performed.
6.  Even when the scheduled period of antibiotic use is completed, the antibiotics should be continued if redness and swelling are still present.
7.  If mastectomy and TE implantation are complicated by uncontrolled atopic dermatitis, TE implantation should be reconsidered, with reconstruction using autologous tissues considered as an alternative. If a patient still wishes to have synthetic-based reconstruction, rigorous preoperative skin care is needed, and skin cultures should be prepared. Depending on the culture results, an anti-MRSA drug should be administered preemptively.

If these precautionary points are taken seriously, they should prevent many of the infections of synthetic materials that are currently seen.

In conclusion, for breast reconstruction with synthetic materials, a major complication of infection should be kept in mind, and patients should be well informed. Rigorous perioperative wound management should be exercised. For patients with risk factors for complications, a surgical approach, such as the use of autologous tissues, should be considered.

## Figures and Tables

**Table1:** Risk factors for postoperative tissue expander infection

Risk factors	Presence of infection	Absence of infection	P value
Age			
<60 years	3 (2.5%)	119 (97.5%)	1
≥60 years	0	23 (100%)	
Obesity			
BMI ≥25 kg/m^2^	1 (3.2%)	30 (96.8%)	0.517
BMI <25 kg/m^2^	2 (1.8%)	112 (98.2%)	
Timing of reconstruction			
Primary	3 (2.8%)	103 (97.2%)	0.564
Secondary	0	39 (100%)	
Radiation therapy			
Preoperative	0	6 (100%)	1
Postoperative^a^	0	2 (100%)	
None	3 (2.2%)	134 (97.8%)	
Chemotherapy			
Preoperative	0	1 (100%)	0.247
Postoperative	1 (8.3%)	11 (91.7%)	
None	2 (1.5%)	130 (98.5%)	
Necrosis at skin margin			
Yes	2 (33.3%)	4 (66.7%)	0.004
No	1 (0.7%)	138 (99.3%)	
Atopic dermatitis			
Yes	1 (50.0%)	1 (50.0%)	0.041
No	2 (1.4%)	141 (98.6%)	

BMI: body mass index ^a^ After implant reconstruction
